# Vitamin D and clinical disease progression in HIV infection: results from the EuroSIDA study

**DOI:** 10.1186/1758-2652-13-S4-O43

**Published:** 2010-11-08

**Authors:** JP Viard, JC Souberbielle, O Kirk, B Knysz, M Losso, J Gatell, C Pedersen, JR Bogner, A Mocroft, JD Lundgren

**Affiliations:** 1 Hopital Necker, Paris, France; 2 Copenhagen HIV Program, University of Copenhagen, Copenhagen, Denmark; 3 Medical University, Wroclaw, Poland; 4 Hospital JM Ramos Mejia, Buenos Aires, Argentina; 5 Hospital Clinic i Provincial, Barcelona, Spain; 6 Odense University Hospital, Odense, Denmark; 7 Medizinische Poliklinik, Munich, Germany; 8 University College London Medical School, London, UK

## Purpose of study

Since 25-hydroxy vitamin D (25(OH)D) deficiency has been associated with higher risk of morbidity and mortality in different settings, this study examined the association between 25(OH)D level and disease progression in HIV-infected patients with prospective follow-up in the EuroSIDA study.

## Methods

A group of 2000 patients were randomly selected from those with stored samples after stratification by region. 25(OH)D levels were measured in a single laboratory from stored plasma samples. The 1985 available 25(OH)D results were stratified into tertiles. Factors associated with 25(OH)D levels and associations of 25(OH) levels with subsequent risk of all-cause mortality, AIDS and non-AIDS events were analysed, using Poisson regression.

## Results

**Figure 1:**
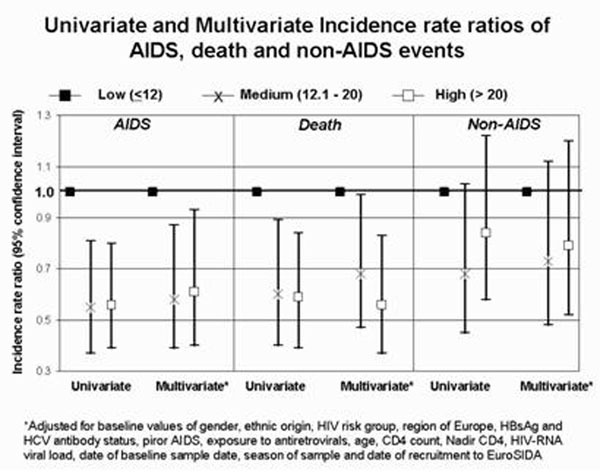


Thirty-six percent of patients had 25(OH) levels below 12 ng/ml, 31,3% between 12.1 and 20 ng/ml, and 32.7% above 20 ng/ml. In a cross sectional analysis, older persons, patients of Black ethnic origin, living outside Southern Europe and Argentina, sampled during winter, and infected with HIV through non-homosexual exposure were at higher risk of having low 25(OH)D levels, while patients receiving protease inhibitors were at a lower risk. Compared to those in the lowest 25(OH)D tertile, those in the medium and high tertiles had a significantly lower risk of clinical progression. Adjusted incidence rate ratios (IRR; see figure 1) for all-cause mortality were 0.68 (95%CI : 0,47-0,99, P=0.045) and 0.56 (95%CI : 0.37-0.8, P=0.009), and for AIDS events were 0.58 (95%CI : 0,39-0,87, P=0.0086) and 0.61 (95%CI : 0.40-0.93, P=0.020), for the medium and high tertiles, respectively. There was a non-significant reduced incidence of non-AIDS defining events in the medium and high tertiles, and a significant lower IRR of non-AIDS related death in the highest 25(OH)D tertile : 0.60 (95%CI : 0.37-0.98, P=0.043).

## Conclusions

This observational study demonstrated that 25(OH)D deficiency is frequent in HIV-infected patients, and is independently associated with a variety of outcomes, reflected by a higher risk of mortality and AIDS events. Whether the relationship between vitamin D deficiency and clinical events is causal should be addressed because of potentially major consequences in terms of public health.

